# Supplementary material for: Content-rich biological network constructed by mining PubMed abstracts
Source: BMC Bioinformatics. 2004 Oct 8;5:147. doi: 10.1186/1471-2105-5-147 (PMC528731; doi:10.1186/1471-2105-5-147)
Supplement: Additional File 2 — The original results of the above study (non-essential files are deleted to keep the file size under the limit set by BMC bioinformatics). [file 1471-2105-5-147-S2.bz2 › chilibotAdditionalFile2/dip10/754ID10782989E18139/html/SSB_SBCB.html]

 


 **SSB** and **SBCB** 
  
Found 13 abstracts in PubMed, retrieved 10.  
 

 What does Google say? 
 PDF only 
| .edu only 

---

**Interactive relationship** (e.g. stimulation, inhibition, etc)

**Stimulatory relationship**- It was shown previously that  **SSB**  can interact with exonuclease I  [ **SBCB** ]  to stimulate the hydrolysis of single stranded DNA.  Ref: 8121810 Nucleic Acids Res, 1994
- We have also demonstrated that  **Ssb**  can stimulate the dRpase  [ **SBCB** ]  activity of exonuclease I  [ **SBCB** ]  using double stranded bacteriophage M13 DNA containing several strand interruptions at incised AP sites.  Ref: 8619028 Radiat Res, 1996
- Escherichia coli single stranded DNA binding protein  [ **SSB** ]  stimulates the DNA deoxyribophosphodiesterase  [ **SBCB** ]  activity of exonuclease I  [ **SBCB** ] .  Ref: 8121810 Nucleic Acids Res, 1994
- We demonstrate here that E. coli  **SSB**  can also enhance the DNA deoxyribophosphodiesterase  [ **SBCB** ]  dRpase  [ **SBCB** ]  activity of exonuclease I  [ **SBCB** ]  by stimulating the release of 2 deoxyribose 5 phosphate from a DNA substrate containing AP endonuclease incised AP sites, and the release of 4 hydroxy 2 pentenal 5 phosphate from a DNA substrate containing AP lyase incised AP sites.  Ref: 8121810 Nucleic Acids Res, 1994
**Neutral relationship**- Interaction of E. coli single stranded DNA binding protein  [ **SSB** ]   **SSB**  with exonuclease I  [ **SBCB** ] .  Ref: 10782989 Biol Chem, 2000
- We have now used the yeast two hybrid system to demonstrate that a protein protein interaction occurs between exonuclease I  [ **SBCB** ]  and  **Ssb** .  Ref: 8619028 Radiat Res, 1996
- Protein protein interactions between the Escherichia coli single stranded DNA binding protein  [ **SSB** ]  and exonuclease I  [ **SBCB** ] .  Ref: 8619028 Radiat Res, 1996
- The origin of this product is here shown to be the result of the exonuclease activity of exonuclease I  [ **SBCB** ]  either added exogenously or present as a trace contaminant of recA protein or  **SSB**  protein preparations, as opposed to endonucleolytic or mechanical breakage.  Ref: 8325876 J Biol Chem, 1993

**Non-interactive relationship** (e.g. studied together, co-existance, homology, etc.)

- E. coli  **SSB**  and exonuclease I  [ **SBCB** ]  form a protein complex as demonstrated by Superose 12 gel filtration chromatography.  Ref: 8121810 Nucleic Acids Res, 1994
- When the E. coli  **ssb**  gene was fused in frame to the DNA activating domain of the GAL4 transcriptional activator and the exonuclease I  [ **SBCB** ]  gene was fused in frame to the DNA binding domain, a functional GAL4 transcriptional activator was produced as determined by growth of yeast on selective medium and the measurement of beta galactosidase activity.  Ref: 8619028 Radiat Res, 1996
